# Addressing global inequities in surgery with the FAIR and CARE principles

**DOI:** 10.1093/bjs/znaf026

**Published:** 2025-03-07

**Authors:** Justin W Collins, Prokar Dasgupta

**Affiliations:** Department of Targeted Intervention, University College London, London, UK; Chair in Robotic Surgery, Department of Immunobiology, King’s College London, Responsible AI UK, London, UK

Surgical training and integrated service delivery often struggles to deliver on the principles of distributive justice, which aims to distribute resources fairly. Distributive justice concentrates on outcomes and aims to identify inequalities in resources and to deliver equity. Globally there are huge disparities in access to surgical expertise. *The Lancet*-commissioned review on Global Surgery 2030^1^ concluded that nearly five billion people do not have access to surgical care. Approximately 94% of individuals in high-income countries have access to surgery compared to only 14.9% in low- and middle-income countries (LMIC). It is estimated that the cost to train the identified shortfall of 2.2 million surgeons and anaesthetists required for LMIC to have parity would be a staggering $350 billion^1^. However, the loss to income in LMIC from premature death and chronic illness related to this lack of surgical intervention is estimated at $12.3 trillion^1^. The Commission’s findings indicate that the human and economic consequences of untreated surgical conditions in LMIC are the number one issue in healthcare, with a greater negative socioeconomic impact than malaria, TB, and HIV combined^1^.

However, disparities are not limited to LMIC and an increasing focus on quality performance reporting following surgery has highlighted disparities in the quality-of-care patients receive in the USA^2^. Research on surgical disparities has identified elements such as race/ethnicity, socioeconomic status, and other demographic factors which correlate with adverse patient outcomes after surgery^3^. As surgical quality improvement programmes proliferate, we must return to one of the central tenets of the National Institute of Health–American College of Surgeons Symposium on Surgical Disparities Research: ‘No quality without access’.

Access to surgical expertise requires awareness of the need to train more surgeons to benchmarked skill levels, agreement on what those benchmarks are and how they are set. Another key element is decision-making in diagnosis, patient selection, treatment, and recovery. Do you have ‘the right patient, for the right surgeon, at the right time in their learning curve’? One of the goals of surgical data science is to use data normalization to standardize metrics of surgical performance to better understand performance and evaluate decision-making^4^. Given the disparities linked to geography and socioeconomic status the challenge then becomes how to make these metrics and training materials accessible, applicable, and affordable to all. Sharing knowledge and objectively defining skills has the added benefit of enabling deliberate practice, which has been shown to shorten learning curves by up to 10 times^5^. However, challenges remain on how to efficiently transfer reusable shared data on expert knowledge of optimized surgical care management and expert surgical performance, while respecting data ownership and delivering secure data environments^6^.

The FAIR and CARE principles are complementary sets of guidelines for data access, retrieval, usability (FAIR) and governance that aims to ensure data are handled in a way that is respectful of all the various stakeholders’ rights and interests (CARE)^7^. In this article we summarize the potential impact of applying these complimentary sets of guidelines in surgical training in a future-looking digitalized setting (see *Table 1*).

**Table 1:** Application of FAIR and CARE principles to surgical training

	Principles	Elements to consider	Compliance and clinical governance
FAIR	Findable	Network development Timely metrics Comprehensive metrics Basic machine-readable descriptive metadata enable human and machine discovery of interesting performance metrics	Resource is uploaded to a secure public repository Metadata are assigned a globally unique and persistent identifier
	Accessible	Accessible in multiple educational settings Recorded content that is retrievable	Accessible and usable in different educational settings Enables a continuum in surgical training between various models and virtual reality/augmented reality settings Uses standard vocabulary Accessible for download or manipulation by humans and is ideally also machine readable
	Interoperability	Shared taxonomy that can be exchanged, interpreted and combined with other data sets by humans and medical devices. Aligned with kinematic data Used in virtual reality/augmented reality settings (metaverse) Used in 3D hydrogel models	
	Reusable	Open-source metrics used in academia, research and training Proper citation must be facilitated and the conditions under which the data can be used should be clear to humans and machines	Enables objective comparison Drives standardization
CARE	Collective Benefit	Inclusive development and innovation of evolving metrics Equitable outcomes	Improved governance and surgeon engagement Stakeholders rewarded with value creation linked to data ownership/contribution
	Authority to control	Recognizing rights and interests related to data ownership, for example patients, trainers, trainees, payees	Data for governance Governance of data
	Responsible	For positive relationships Expanding capability and capacity Sharing insights to aid progress	
	Ethics	Minimizing harm and maximizing benefits For future utilisation of metrics	Open by default Distributive justice

Standardization of data reporting on surgical technique, anatomy, and outcomes enables data analysis to identify areas for further optimization, resulting in continuous improvement loops between standardization and optimization^8^. Standardized training can also reduce variables in data analysis and aid the use of artificial intelligence (AI) applications in surgery^6^. An approach to help this workflow in digital surgery is to standardize training *via* train the trainer programmes^9^, then develop networks *via* telepresence services that could eventually include telesurgery. Data can then be collected *via* the network and linked to outcome data incorporated into the analysis, resulting in continuous incremental improvements that can be identified and shared within the network(s)^6^. The transferability and adoption of shared knowledge and insights from recurring hazardous situations or near misses has been successfully utilized in other high-reliability organizations such as the aviation industry to improve decision-making in common critical situations^10^. Having globally accepted reference metrics for surgical knowledge and skills will enable more training to be delivered in reusable digital platforms such as virtual reality/augmented reality and disseminated online or *via* social media platforms in ways that can be more easily monitored for quality assurance and clinical governance^4^. An approach to sharing data on agreed and optimized surgical training metrics that shortens learning curves for surgical trainees in LMIC would give access to expert knowledge while also improving efficiencies in skills training for surgical trainees^5^. However, LMIC require substantial investment to improve digital infrastructure and be equal partners in responsible data sharing. There will also be ethical, legal, and social considerations that will need to be resolved for safe and equitable sharing of surgical data.

The FAIR principles stand for Findable, Accessible, Interoperable, and Reusable. These principles focus on the characteristics of data that make it easier to share, while avoiding issues related to power dynamics or historical contexts. The FAIR principles have been used in multiple settings and have potential to guide data sharing in surgery. The CARE principles stand for Collective Benefit, Authority to Control, Responsibility, and Ethics. These principles are people- and purpose-oriented, and focus on how data can be used to advance Indigenous innovation, self-determination, and governance. The current identified needs for surgical training that could be improved with promotion of these principles include:

1. An awareness of the importance and benefits of training.
2. Agreement on training performance metrics.
3. A current lack of objective metrics that enables deliberate practice with personalized performance feedback.
4. Access to standardized training (current limitations include time, simulation labs, MedTech devices).
5. Affordable approaches to training in different settings and networks that enable scalability.
6. Lack of interoperability from data-collection devices used in training.
7. Improved engagement and alignment of benchmarks with surgical societies, institutions, and individual surgeons.

International collaboration and shared data agreements are urgently needed to help identify resource inequalities and reduce global inequities in both access and quality of surgery. To achieve this, societies, regulatory bodies, and industry need to recognize this important issue and align educational goals. To reduce costs by sharing knowledge, delivering more training locally, utilizing telepresence services, and making reusable content such as educational videos and synthetic training models more widely available. This complex integrated process can be guided by the complementary FAIR and CARE Principles. Although the FAIR principles are about making it easier to share and reuse resources, the CARE principles ensure that resources, including data, are used ethically and equitably. Success will be measured by our outcomes.
